# Some Cases of Hemiplegia

**Published:** 1904-09-17

**Authors:** 


### Hospital Clinics.

### SOME CASES OF HEMIPLEGIA.

Dr. R. T. Williamson[1] records three instances which illustrate the diagnostic value of a hemiplegia of gradual onset in the case of cerebral tumour. When with the hemiplegia there are vomiting, headache, and double optic neuritis, the position is a comparatively simple one. Provided that cerebral abscess can be excluded, such a combination of events points in no uncertain or hesitating manner to a diagnosis of cerebral tumour. But it is necessary to recognise that the absence of these last-mentioned symptoms, in face of a hemiplegia which slowly and gradually extends so that it may be weeks or months before it is complete, does not invalidate the diagnosis of tumour. Weakness commencing in one hand or foot, then gradually involving all the movements of the limb, and afterwards affecting the other limb of the same side suggests in the strongest possible manner the presence of a tumour either in the motor cortex or the motor white matter of the cerebral hemisphere opposite to the paralysed area. In none of the three cases which Dr. Williamson records was there optic neuritis; in one vomiting and headache were entirely absent; in a second vomiting and headache were early symptoms; whilst in the third neither of these conditions existed until nine weeks after the onset of the signs of paresis and four weeks before death. In only one of the cases were there any unilateral convulsions. The slow onset of the paralysis is a valuable clinical point in the diagnosis of tumour as distinct from embolism, hæmorrhage and thrombosis. At the same time it must be remembered that a rapid development of hemiplegia does not exclude tumour from the diagnosis. In many instances it has been known that a tumour has existed for some considerable period without producing any signs of paralysis and then these have suddenly occurred. Presumably in these instances the fibres of the motor tract have not been involved until, in consequence of a sudden extension of the tumour or of a hæmorrhage in its neighbourhood, pressure has been exerted on them with resulting rapid development of hemiplegia.

In a clinical lecture on several cases of hemiplegia Dr. Norman Moore[2] places much stress upon the fact that when this condition is due to syphilis there are nearly always signs that the paralysis is not strictly limited to one side of the body. The cerebral effects of syphilis are usually in the form of scattered lesions with a corresponding widespread character in the paralyses. Indeed when symptoms exist in this form the fact is strongly suggestive of cerebral syphilis, and is thus of material assistance in diagnosis and treatment. The explanation, of course, is that in a very large number of cases the changes in the brain tissue are secondary to vascular disease in the form of scattered degenerations and thickenings in the walls of the arteries. Such changes produce patches of softened or ill-nourished brain tissue, and the degree of this mal-nutrition may vary at various times. Hence the symptoms of cerebral syphilis are not only apt to be spread over a large area but they may vary in their intensity at different times. This fluctuating character of the symptoms is a further help to diagnosis. It must also be remembered in relation to prognosis. In an ordinary case of hemiplegia not due to syphilis but caused, for example, by a cerebral hæmorrhage the condition of the patient will hardly improve in any substantial degree after the first two to three weeks. But in syphilitic cases the position is different. There is a much better prospect of improvement even though a not inconsiderable amount of time elapses before this appears. Mercury and iodide of potassium must therefore be perseveringly employed, even though there appears to be little or no response to the treatment, or the patient perhaps develops further evidences of the disease. The same contrast exists between syphilitic and non-syphilitic hemiplegia in reference to the mental functions. Such serious conditions as aphasia and amnesia may be found in the syphilitic patient, and yet he may entirely recover from them, whereas an elderly man with similar symptoms, the result of cerebral hæmorrhage, will in all probability be permanently prejudiced in his mental ability and power. These considerations indicate and emphasise how necessary it is to recognise syphilitic disease of the brain when it occurs, and to prosecute the appropriate treatment with unfailing resolution.

Dr. T. R. Bradshaw[3] contrasts two cases of hemiplegia, the one in a man of 25 years, the other in a woman *æt.* 22. In each case the paralysis was of sudden onset. That in itself suggests a vascular lesion—hæmorrhage, thrombosis, or embolism. The last-mentioned necessitates a source from which a clot may be dislodged, generally cardiac valve disease. The two former are always associated with arterial degeneration — hæmorrhage with a degeneration that tends to rupture, thrombosis with arterial thickening and narrowing of the calibre of the vessel, the final closure being completed by the formation of a clot *in situ*. The two main causes of arterial disease are senile degeneration and syphilis. Hence hemiplegia in a patient under 40 years of age is most probably due either to valvular disease of the heart or to lues venerea. In the first of the two cases recorded by Dr. Bradshaw the patient—a man of 25 years—had contracted a venereal sore one year before the onset of the paralysis. No secondary symptoms were observed, but after three weeks of severe occipital headache he suddenly fell to the ground and was found to be paralysed on his right side. An hour or so later the paralysed parts were convulsed and this condition lasted some 24 hours. Speech was interfered with but there was no loss of consciousness. After three months he had regained a sufficient amount of power to permit him to walk with the aid of a stick. In addition to the history of infection and the age of the patient there are two other facts in the record which support the diagnosis of syphilis. The one is severe headache, which, coming on in a young adult previously free from any such condition, should always suggest syphilis. The other is the retention of consciousness at the time of the occurrence of paralysis. This is strongly against the diagnosis of hæmorrhage. It also opposes the theory of embolism in which, unless the vessel occluded is a very small one, consciousness is usually lost. Hence there remains only thrombosis, which, as already pointed out, is generally dependent in young adults on syphilitic disease of the arteries, and in which, as a matter of experience, hemiplegia frequently occurs without any accompanying development of coma. History, age, pre-existing severe headache, and paralysis developing without loss of consciousness, are all features in the case which favour the diagnosis of syphilis. It is of interest to note the sudden development of the hemiplegia. On theoretical grounds it might have been supposed that the onset of a paralysis dependent on thrombosis must needs be gradual. But clinical experience shows that this is not necessarily the case. The gradual narrowing of the calibre of the artery as a result of syphilitic thickening of its wall may indeed disturb the nutrition of the brain in the area supplied by the artery, and may thus give rise to giddiness or other cerebral symptoms which thus precede and perhaps foretell the onset of the paralysis. But the final closure of the artery by thrombosis is a relatively sudden event, and hence a hemiplegia of sudden development does not negative the view that thrombosis is its proximate cause. Two other features of the case now in question are noteworthy, namely, the occurrence of hemiplegia so early as 12 months after a primary syphilis, and the non-observance of any secondary symptoms. With reference to the first of these it used to be considered that the effects of syphilis on the nervous system were among the late or so-called tertiary phenomena of the disease. But a considerable body of evidence has been produced to modify this view. More particularly Mr. Jonathan Hutchinson [4] has recorded a considerable number of cases of early paraplegia after syphilitic infection. In several of these the interval was less than a year and in some it did not exceed six months. Failure to observe secondary symptoms is not so very uncommon, and in Dr. Bradshaw's case the patient was said to have been under treatment for seven months, and thus there may have been a more or less complete suppression of the secondary evidences of the disease.

The second patient on whom Dr. Bradshaw's lecture was based was a woman of 22 years. There was a history of rheumatic fever and of scarlet fever, and the loss of power in the left arm and leg occurred suddenly. Naturally these facts, together with the age of the patient, raised the presumption of organic heart disease, probably mitral stenosis, as this is the lesion which most commonly gives rise to embolism. But, on examination of the heart, there was found merely a soft systolic murmur heard at the apex, and little or no evidence of cardiac hypertrophy. Such a state of matters threw some doubt on the diagnosis of embolism, and this was further confused by a history of headache which was reported as troublesome for several days prior to the paralysis. However, after a few days there was evidence of febrile disturbance, the temperature following a hectic type. In these circumstances, a suspicion of infective or ulcerative endocarditis was entertained, and this was strengthened by the discovery of an enlarged spleen. Finally, all doubt was removed by the discovery of streptococci in the blood. A diagnosis of hemiplegia, due to infective endocarditis, carries necessarily a very much more serious prognosis than a hemiplegia due to syphilis or to simple embolism, as the primary disease is one of the most fatal with which the physician has to deal. Recovery, however, is not quite unknown, and the prognosis improves if the case proves to be a long continued one. Quinine in full doses is perhaps the most useful medicinal agent. In some cases antistreptococcic serum has also been tried, but without any very satisfactory results.

In reference to the statement that the occurrence of paralysis due obviously to some cerebral event in a patient under 40 years of age must mean either embolism or syphilitic thrombosis it is well to note that this proposition, like many others in the field of clinical medicine, is not an absolute one. There are many well authenticated instances in which hæmorrhage has been the cause of coma and paralysis in quite young people. Dr. T. M. Pearce [5] has recorded such an instance in a lad of 19 years and has also drawn attention to a collection by Edward Sanders of 94 cases, of which seven occurred in patients under 12 months of age and 14 before the age of 20 years. In another example [6] the patient, a young woman, died from a hæmorrhage into the cerebellum.

[1] Pract., Sept., 1904. [2] Clinical Journ., July 6, 1904. [3] Lancet, July 16, 1904. [4] Archives of Surg., Vol. VII., *et seq.* [5] Lancet, Nov. 15, 1902. [6] Lancet, May 18, 1895.

